# Symbionts as Major Modulators of Insect Health: Lactic Acid Bacteria and Honeybees

**DOI:** 10.1371/journal.pone.0033188

**Published:** 2012-03-12

**Authors:** Alejandra Vásquez, Eva Forsgren, Ingemar Fries, Robert J. Paxton, Emilie Flaberg, Laszlo Szekely, Tobias C. Olofsson

**Affiliations:** 1 Department of Laboratory Medicine, Medical Microbiology, Lund University, Lund, Sweden; 2 Department of Ecology, Swedish University of Agricultural Sciences, Uppsala, Sweden; 3 School of Biological Sciences, Queen's University Belfast, Belfast, United Kingdom; 4 Institute for Biology, Martin-Luther-University Halle-Wittenberg, Halle (Saale), Germany; 5 Department of Microbiology, Tumor and Cell Biology (MTC), Karolinska Institutet, Stockholm, Sweden; University of Hyderabad, India

## Abstract

Lactic acid bacteria (LAB) are well recognized beneficial host-associated members of the microbiota of humans and animals. Yet LAB-associations of invertebrates have been poorly characterized and their functions remain obscure. Here we show that honeybees possess an abundant, diverse and ancient LAB microbiota in their honey crop with beneficial effects for bee health, defending them against microbial threats. Our studies of LAB in all extant honeybee species plus related apid bees reveal one of the largest collections of novel species from the genera *Lactobacillus* and *Bifidobacterium* ever discovered within a single insect and suggest a long (>80 mya) history of association. Bee associated microbiotas highlight *Lactobacillus kunkeei* as the dominant LAB member. Those showing potent antimicrobial properties are acquired by callow honey bee workers from nestmates and maintained within the crop in biofilms, though beekeeping management practices can negatively impact this microbiota. Prophylactic practices that enhance LAB, or supplementary feeding of LAB, may serve in integrated approaches to sustainable pollinator service provision. We anticipate this microbiota will become central to studies on honeybee health, including colony collapse disorder, and act as an exemplar case of insect-microbe symbiosis.

## Introduction

Symbiosis is common in nature, in which symbionts as commensals or mutualists evolved to benefit each other. Culture-independent studies of the human microbiota identified recently a complex symbiotic environment with more than 1,000 bacterial phylotypes representing more than 7,000 strains [1]. The composition of this microbiota has been suggested to be a result of a highly coevolved symbiosis and commensalism influenced by nutrition, physiology and immunological factors [2], [3].

The insect gut has been described as the greatest unexplored reservoir of microbiological diversity [4]. Ryu and colleagues [5] established the importance of the normal flora in the fruit fly gut in order to sustain health. This small microbiota was sufficient to suppress growth of pathogens. While insects harbour a smaller number of symbionts compared to humans they may be even more important [6]. Studies have shown that symbiosis between social insects and microbial species are often highly coevolved [7] and that these symbionts are evolutionary shaped distinctly from the forces acting upon symbionts of solitary organisms, which normally lack a homeostatic fortress environment [8].

Lactic acid bacteria (LAB) are found as commensals within humans, insects and animals [9]. They confer an important bacterial group for the food industry and the fermentation of dairy products. In addition, strains within LAB are also generally recognized as safe (GRAS) food grade microorganisms and employed as probiotics bestowing human health [10], [11]. Genera within LAB are functionally related by phenotypic characteristics [12] and considered as beneficial organisms commonly found as both exogenous and endogenous microbes in healthy individuals. LAB found within humans and animals as commensals are known to protect their hosts via antimicrobial metabolites and modulation of host immune response [13], [14]. One of the most important genus within LAB is *Lactobacillus*, which is continuously under taxonomic discussion and includes at present 175 listed species [15].

We have discovered a rather special symbiotic lactic acid bacterial (LAB) microbiota within the honey crop of the Western honeybee *Apis mellifera* [16]. The crop is a central organ in the honeybee's food production between the oesophagus and ventriculus, used for collection and transport of nectar to hive. The crop microbiota of *A. mellifera* is composed of 13 bacterial species within the genera *Lactobacillus* and *Bifidobacterium* [16], [17], [18] and it plays a key role in the production of honey [16] and bee-bread [19], long term stored food for both adult honeybees and larvae. Our recent studies on the subspecies of *A. mellifera* have also demonstrated that the LAB microbiota is consistent across its native and introduced range [17].

Metagenomics has been used to identify a rich diversity of microbes within honeybees afflicted by Colony Collapse Disorder (CCD) [20], including emergent pathogens (i.e. *Nosema ceranae* and viruses) [21], while recent studies have picked up novel bacterial genera within the intestinal tract of bees by culture independent methods [20], [22], [23]. Some of these may comprise important symbionts for the maintenance of bee health; however, these descriptive methods do not inform on the functional role or importance of the bee crop microbiota or of individual symbionts within this niche.

We have demonstrated by both *in vitro* and *in vivo* studies that the LAB microbiota in *A. mellifera* inhibit one important honeybee pathogen, the bacterial brood pathogen *Paenibacillus larvae* that is the cause of the brood disease American foulbrood (AFB) [24]. In the current study we investigate if the LAB microbiota is consistent in all nine recognized honeybee (Apini) species plus stingless bee species (Meliponini), a phylogenetically close taxon that, like honeybees, are eusocial, live in colonies comprising one queen and 100's to 10,000's of workers, store large quantities of honey and bee bread and are managed commercially or exploited by ethnic groups across the tropics.

Functional characterization of the endogenous crop microbiota is essential in providing insights for the understanding of its role for bee health and disease. Here we explore the diversity, maintenance and dynamics of LAB in the honey crop and the pivotal role that they play in bee health, with major implications for research on bee decline and sustainable pollinator management.

## Results

### LAB diversity

**Figure 1:**
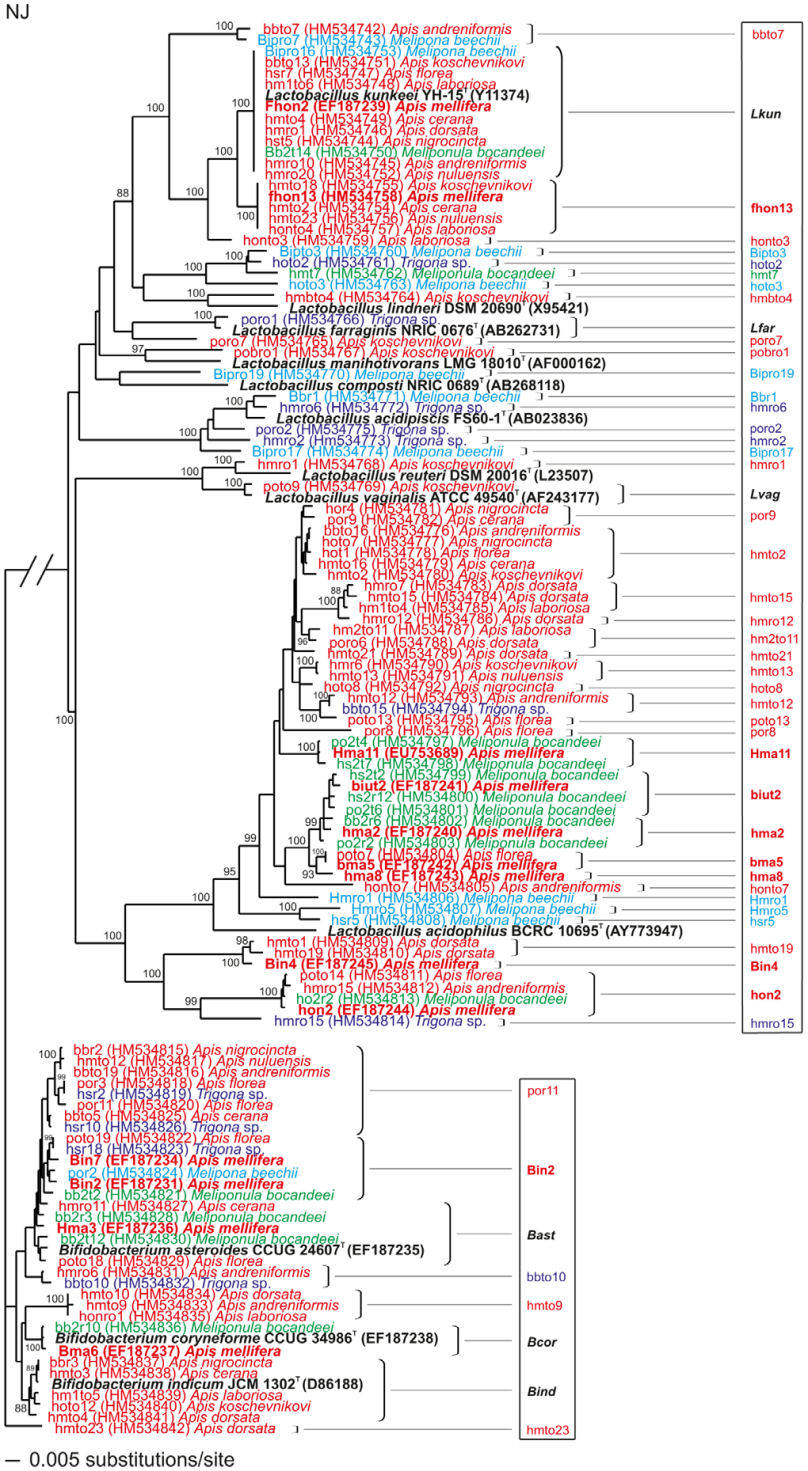
Phylogenetic tree of LAB in bee crops. Phylogenetic tree based on a distance matrix of positions 56–1470 (*Escherichia coli* numbering) in the 16S rRNA gene of *Lactobacillus* and *Bifidobacterium* spp. bacteria from *Apis* species (red) and the stingless bees *Melipona beechii* (turquoise), *Meliponula bocandeii* (green) and *Trigona* sp (blue). Previously characterized bacterial phylotypes from *Apis mellifera* are in bold print (red). Bacterial type strains are in bold characters (black). Phylotypes in the framework (right) represent different clusters belonging to a characterised or possibly novel species. *Bifidobacterium* group is the out-group. Bar: 5 base pair changes. 16S rRNA gene sequences deposited in GenBank HM534742–HM534842 (in parenthesis).

All 9 *Apis* and the 3 Meliponini species studied possess a similar microbiota, comprising together approximately 50 novel LAB species in the genera *Lactobacillus* and *Bifidobacterium* (Figure 1), using the threshold used to define a bacterial species based on rRNA gene sequencing of <97% similarity [25] (<98.5% is often found to imply possibly novel species).

One particular LAB was common and dominated the microbiota of *Apis* spp.: *L. kunkeei* (44% of 750 isolates were *L. kunkeei*). However, many *Lactobacillus* and *Bifidobacterium* were found to be common across bee species (Figure 1), including *L. kunkeei* in stingless bees from Central America (*M. beecheii*) and Africa (*M. bocandei*), though not in *Trigona* sp. from Borneo and Thailand, where it is sympatric with five native *Apis* species. From our previous studies [16], [19] we know that bees add the LAB microbiota to their honey and corbicular pollen. In this study, we found the highest concentration of viable LAB (10^8^ per gram honey) in Nepalese honey of *A. laboriosa* and similar quantities in *A. mellifera* honey from Africa (Table 1).

**Table 1:** Lactic acid bacterial counts in social bees.

Bee species	honey crop	honey (g^−1^)	bee pollen (g^−1^)	bee bread (g^−1^)
*Apis mellifera* a b	10^6^	10^7^	10^8^	10^6^
*Apis nuluensis* b	10^6^	ND^c^	10^6^	ND^c^
*Apis nigrocincta* b	10^7^	10^5^	10^9^	10^5^
*Apis koschevnikovi*	10^5^	10^6^	ND^c^	10^5^
*Apis cerana*	10^6^	10^5^	10^6^	10^5^
*Apis andreniformis* b	10^3^	10^4^	10^6^	10^4^
*Apis florea* b	10^6^	10^2^	10^6^	ND^c^
*Apis laboriosa* b	10^6^	10^8^	ND^c^	10^9^
*Apis dorsata* b	10^5^	ND^c^	10^6^	ND^c^
*Melipona beecheii* b	10^7^	10^5^	10^6^	10^6^
*Meliponula bocandei* b	10^7^	10^3^	10^6^	10^7^
*Trigona sp.* b	10^5^	10^4^	10^7^	10^4^

a *Apis mellifera* bees collected in Kenya from the subspecies *A. m. scutellata* and *A. m. monticola*.

b Wild colonies.

c ND = Not determined.

**Legend.**

Viable counts in colony forming units (cfu) of lactic acid bacteria cultivated from honey crops, fresh honey, bee pollen and bee bread. All samples were cultivated on LAB selective media immediately after sampling or after chilled transportation.

### Ontogeny and maintenance of LAB

The results were clear-cut; at eclosion, crops were empty and devoid of LAB; but within minutes post-eclosion the microbiota builds up gradually by trophallactic exchange with nestmates (Table 2). *L. kunkeei* was found to dominate the crop microbiota at all sampling occasions. Only six honeybee crop LAB members were found during the trial. Honeybees kept in solitary confinement from eclosion (n = 10) retained sterile crops.

**Table 2:** Presence of LAB in callows.

LAB	At eclosion^a^	1 h	24 h	3 days	1 week	2 weeks
Bma5	-	-	-	1	2	3
Hma8	-	1	-	-	-	1
Biut2	-	-	-	-	-	-
Hma2	-	-	-	-	-	-
Hma11	-	-	-	-	-	-
Hon2	-	-	-	-	-	1
Bin4	-	-	-	-	-	-
Fhon13	-	-	-	-	-	-
Fhon2	-	17	6	16	13	9
Bma6	-	-	-	-	-	-
Bin7	-	-	-	-	-	-
Hma3	-	-	1	-	-	1
Bin2	-	-	-	1	1	-

a Displayed numbers represent randomly picked isolates.

**Legend.**

Ten to 30 colonies were randomly picked from agar plates containing 30–300 colonies each, and re-cultivated for purity (isolates). The re-cultivation and identification of LAB were performed as previously described [16].

**Figure 2:**
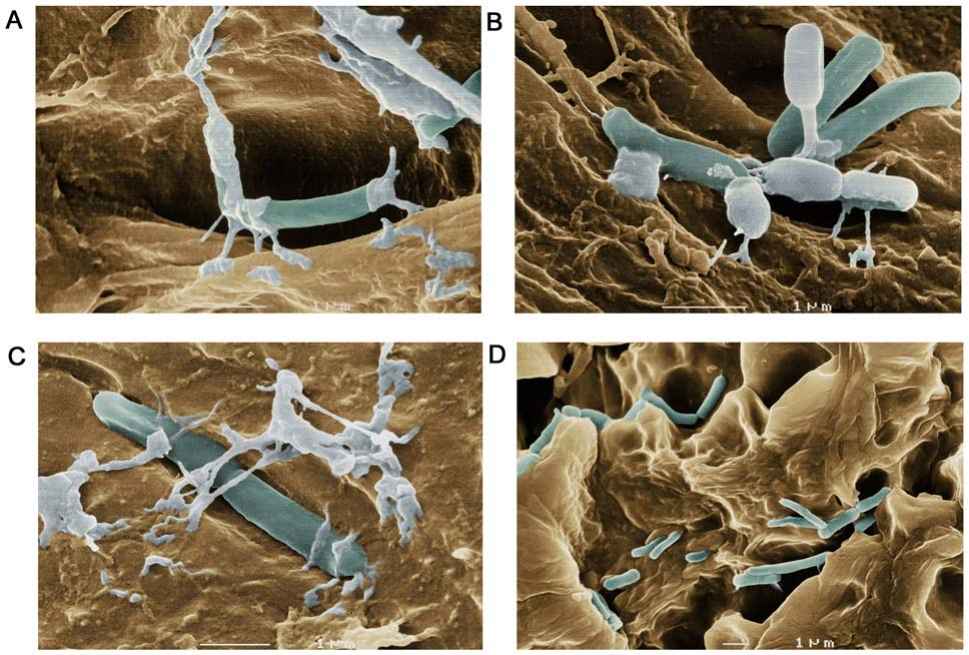
*In vitro* visualization of lactobacilli. *In vitro* visualization of lactobacilli attached to the wall of a honeybee crop using SEM. **A**, **B** and **C** show different areas of a honey crop at similar magnification with visible attachment structures resembling extracellular polymeric substances (EPS). **D** shows a larger part of the crop with attached bacteria. Photographer Lennart Nilsson.

**Figure 3:**
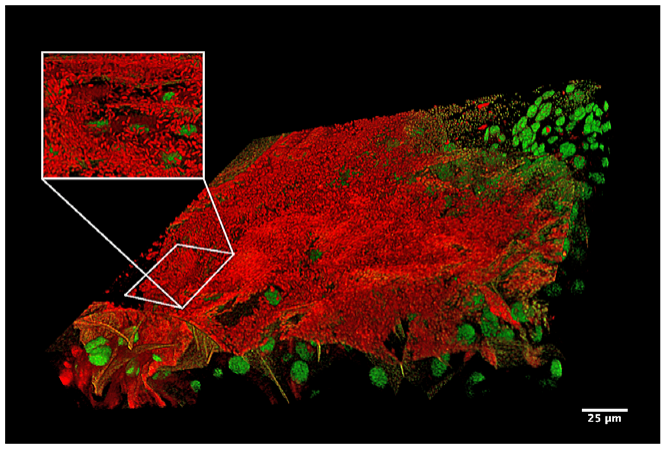
*In vivo* visualization of LAB biofilm. The red fluorescence shows live-stained bacteria in a LAB biofilm attached to a honey crop. The green fluorescence shows the nuclei of the honeybee crop cells. The visualized tissue shows a projection of 112 confocal z-sections (through a 37.6 µm z-depth, covering a xy-area of 246×246 µm).

The LAB microbiota remains viable within bees and added in high concentration to their food products (Table 1) as the symbionts detach from their niche the honey crop. We observed by *in vitro* and *in vivo* investigations of the LAB symbionts with SEM and fluorescence microscopy that the microbiota form biofilms and networks (Figure 2 and Figure 3; Video S1 and Video S2) by which they attach to the wall of the crop using structures resembling extracellular polymeric substances (EPS) (Figure 2).

### Functional characterization

The microbial composition of 15 flowers frequently visited by *A. mellifera* in Sweden was investigated (Table S1). In total sixty transient microorganisms were isolated from flowers and identified to the species level (Accession nr: JN167926-JN167985). We tested the inhibition properties of all honey crop LAB grown individually and together against the 55 bacterial strains and 5 yeast strains isolated from flowers. There was a clear overall inhibition of all transient flower microorganisms by single members of the LAB microbiota in the honey crop (Table S1). *L. kunkeei*, the most common species in all bees (Figure 1), was also the most potent, inhibiting all tested microorganisms.

**Figure 4:**
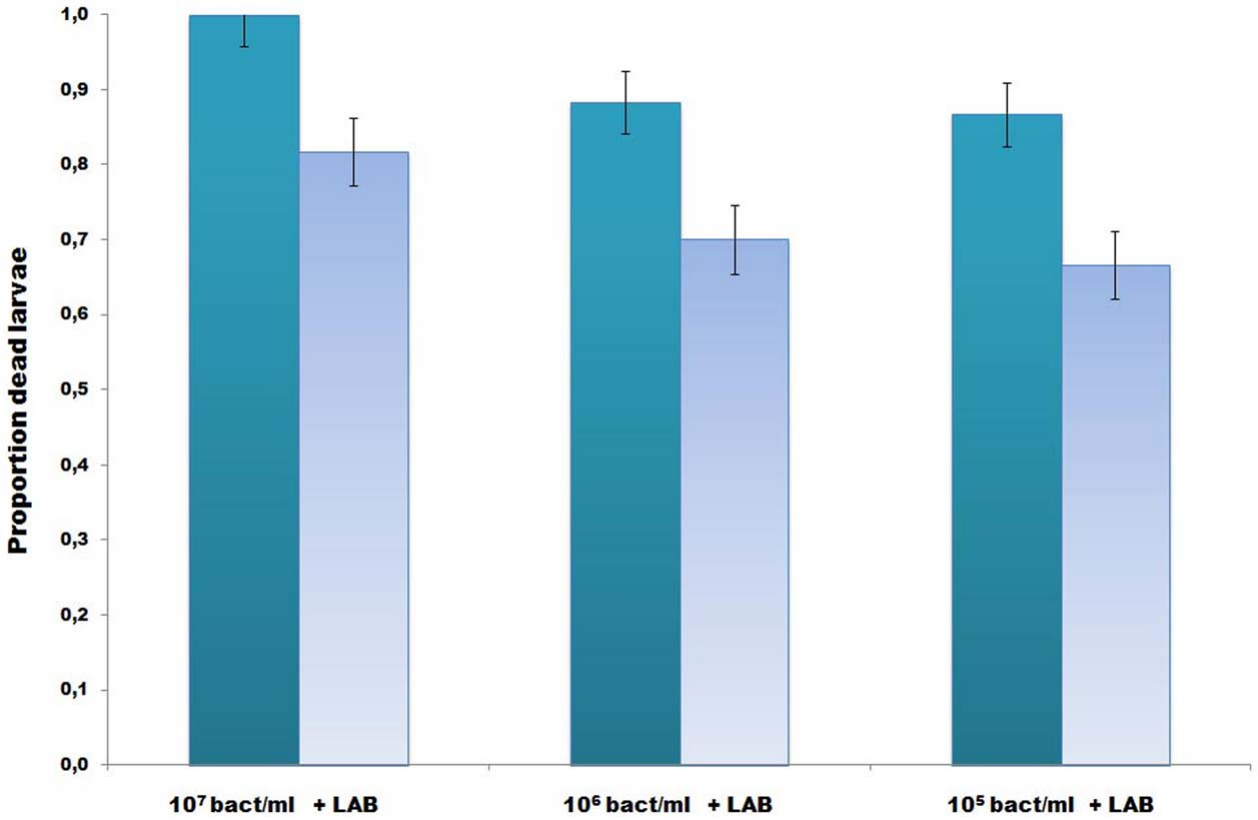
LAB rescue of honeybee larvae from European Foulbrood. Proportion dead larvae in both replicates (total number dead after 21 days). Data presented as a mean for the three groups fed *M. plutonius* (10^7^, 10^6^ and10^5^ bacteria ml^−1^), and the 3 groups fed *M. plutonius* and LAB. Irrespective of infectious dose, the overall effect from the LAB supplement was a significantly reduced mortality in the treated groups. Mortality in the uninfected control groups was <7%.

The LAB microbiota partly inhibited the bee pathogen *M. plutonius in vivo* (Figure 4) and totally *in vitro*, *L. kunkeei* and the thirteen LAB together showed the best inhibition properties. The overall effect of adding the LAB mixture to bee larval food was a significant reduction in the number of dead larvae (Figure 4, X^2^_4_ = 24.27, 2-tail P: <0.001). The total mortality of the uninfected control groups (with or without LAB supplement) was <7% in both replicates. The results demonstrate that addition of LAB to the food of young honeybee larvae exposed to *M. plutonius* decreases the number of larvae succumbing to EFB infection.

### LAB and antibiotics

*In vitro* culturing of the 13 individual LAB members with two antibiotics (oxytetracycline and tylosin) used in apiculture to control bacterial diseases AFB and EFB demonstrated high sensitivity of all LAB members to Tylosin, the most recently employed antibiotic within apicultural practices in the USA [26]. Nevertheless, strains *L. kunkeei* Fhon2 and *Lactobacillus* Fhon13, Hma11, Hma8 and Hon2 showed resistance to oxytetracycline.

### Accession numbers

The 16S rRNA gene sequences generated in this study are available from GenBank under the accession numbers: HM534742–HM534842, JN167926–JN167985 and JN689233.

## Discussion

Our results reveal one of the largest collections of novel species from the genera *Lactobacillus* and *Bifidobacterium* ever discovered within a single insect. A detection of ca. 50 novel species within these bacterial genera will make a huge impact in their current taxonomy. The findings of *L. kunkeei* as common symbionts with *Apis* and stingless bees highlight the importance of this organism. We have previously shown the consistent presence and dominance of this lactobacilli, in our studies of *A. mellifera* (25% of 158 isolates in Sweden [16], [17], [19]; 40% of 42 isolates in USA [18]; 28% of 50 isolates in Africa [17], and now in all *Apis* spp. and in the stingless bees. The most recent common ancestor to honeybees and stingless bees has been dated to >80 million years ago [27], suggesting that the *L. kunkeei*-dominated LAB flora is an ancient apine bee association. But invariance in *L. kunkeei* 16S rRNA gene sequences across host species and geographic locality (Figure 1) suggest possible horizontal transfer of LAB members between host species. However, in Borneo the five sympatric *Apis* spp. forage together with *Trigona* spp. on the same flowers but *L. kunkeei* was not found in any *Trigona* spp. investigated, arguing against horizontal transfer.

Our investigation shows how this microbiota is acquired (Table 2) and maintained within bees (Figure 2 and Figure 3; Video S1 and Video S2). Interestingly, LAB builds up gradually by trophallactic exchange with nestmates and *L. kunkeei* Fhon2 was found to dominate the crop microbiota at all sampling occasions again reflecting its importance (Table 2). However, that only six honeybee crop LAB members were found during the trial may reflect either the disadvantage for bacterial counting of using viable counts that display the dominant bacteria or the numerical variation across seasons. We know from our previous studies that the LAB members in the crop vary numerically across seasons with the flowers visited by bees and with the health status of bees [16]. On the other hand, we know that the microbiota is also rather consistent across *Apis* species. At first sight, it is surprising how this microbiota is maintained within the honey crop, with the extensive ebbing and flowing of sugars, enzymes, water and the constant invasion of foreign microbes following ingestion of flower nectar during foraging. Visualization of the crop and attached LAB reveals how this microbiota is conserved (Figure 2 and Figure 3; Video S1 and Video S2) in networks and biofilms. The property of biofilm formation is known in LAB species that resides in the human gut and vagina [28], [29], [30]. In addition to the well-described characteristic of LAB to produce exopolysaccharides, other likely mechanisms in biofilm formation and adhesion include the production of proteins, carbohydrates, enzymes, nucleic acids, lipids or membrane bound receptors. Exopolysaccharides are the main component in extracellular polymeric substances (EPS) and when, secreted into the environment, provide protection to bacteria; they are also involved in host colonization and cellular recognition [31]. It has been suggested that exopolysaccharides produced by food grade organisms (GRAS), in particular LAB, may confer health benefits in humans [32], [33], [34]; the same may be true for honeybees. The complexity of attachment and biofilm formation by this symbiotic community comprise yet unknown mechanisms of action. These may include membrane properties of the symbionts to avoid rejection by their host, as well as the production of potent antimicrobial substances.

During foraging, foreign microbes are introduced into bees and to their colony through their honey crop, with collected nectar, and through pollen (Table S1). When a flower blooms for the first time, its nectar and pollen are sterile but eventually become invaded by airborne microorganisms and microbes from insects. The composition and numbers of this transient microbiota may vary with time but also with flower type, visiting insects, temperature and the nutritional composition of the pollen and nectar. LAB members in the crop vary numerically [16] but are consistent within the same *Apis* species [17]. LAB diversity could be explained by variation in nutrient content of different nectars and pollen and also by variation in the microbes to which flowers are exposed. Transient floral microbes may trigger the growth of resident LAB microbiota in bees and their production of antimicrobial substances, a mechanism known for LAB strains in other niches (e.g. *Lactobacillus reuteri* when producing reuterin [35], [36], [37].

We raised the hypothesis that honeybee LAB possess antimicrobial properties against microorganisms present in nectars and on pollen in order to defend their niche (the honey crop) and prevent spoilage of honey and bee bread during their production, which may take from days to weeks. Our results show a preliminary overall inhibition of transient environmental microbes found in flowers (Table S1). Once again, *L. kunkeei*, the most common species in all *Apis* bees (Figure 1), was highlighted as the most potent, inhibiting all tested microorganisms. This bacterium was originally described as a wine-spoiling organism since it inhibited yeast in wine production [38], [39]. Interestingly, the extent of inhibition by single LAB members varied considerably with test microbes. Yet the LAB microbiota seems to work in a synergistic matter [24] (Table S1); they produce antimicrobial agents including common organic acids, proteins, peptides, enzymes, and bacteriocins that we are currently characterising.

The LAB microbiota of the *A. mellifera* honey crop is added by bees to their brood food and corbicular pollen and is important in the production of honey and bee-bread [16], [19]. It is well known that species within *Lactobacillus* and *Bifidobacterium* inhibit pathogens; they have been used for centuries in food preservation to prevent microbial spoilage [40]. Commercial probiotic products for human consumption with viable LAB contain about the same quantity (10^8^) of mostly one single LAB g^−1^ product [41], [42]. We hypothesise that the resident strains of *Lactobacillus* and *Bifidobacterium* in honey could function in a similar way as LAB for food preservation or as a defence against microorganisms invading humans.

Honey collected from managed or wild colonies of *Apis* spp. or stingless bees has been independently regarded as a therapeutic agent by many cultures throughout history, from the Maya in Mexico to the Pharaohs in Egypt [43], possibly reflecting beneficial effects of the viable honeybee microbiota when consumed or applied on wounds. It is feasible to believe that fresh honey represents a naturally occurring probiotic product, one with a great diversity and concentration of LAB species (Table 1) that may reflect a myriad of beneficial properties of every specific LAB member in the honey crop.

We believe that LAB antimicrobial mechanisms have evolved in synergy with bees to defend themselves and their hosts from environmental threats such as microbes found in nectars and pollen, and possibly for defence against specific honeybee pathogens. This ancient symbiotic relationship between LAB and bees seems to be of great benefit for bees and may be referred to as colonization resistance. The same phenomenon described for the normal flora in the fruit fly gut [6]. Honeybee brood are fed bee-bread containing viable LAB and their antimicrobial substances. Thus, our results strongly suggest that LAB linked to the honeybee crop have important implications for honeybee pathology, particularly for bacterial brood diseases such as AFB and EFB. Honeybees are considered to have only about a third of the innate immune genes compared to other insects [44], [45]. In addition to social defences that accrue to social insects [46], individual honeybees may also benefit from their LAB symbionts, which are probably of great importance in pathogen defence, possibly further reducing dependency on the innate immune system.

In order to secure honeybee pollination services, *A. mellifera* beekeepers replace harvested honey by feeding sugar solutions, occasionally mixed with antibiotics for prophylactic control of honeybee-specific bacterial diseases of bee brood such as AFB and microsporidia [47]. It is known that LAB antibiotic susceptibility varies [48], [49]. *In vitro* culturing of the 13 *Apis* individual LAB members with two antibiotics used in apiculture (oxytetracycline and tylosin) demonstrated high sensitivity of all to Tylosin, the most recently employed antibiotic within apicultural practices in the USA [26]. Nevertheless, strains *L. kunkeei* Fhon2 and *Lactobacillus* Fhon13, Hma11, Hma8 and Hon2 showed resistance to oxytetracycline that may reflect the extended use of this antibiotic in apiculture or their long-term exposure to environmental microbes from the surrounding environment that produce similar substances. The negative effects on honeybee health from damaging the honey crop microbiota by the use of these antibiotics need to be investigated further.

### Perspectives

The economic value of commercial honeybee pollination is estimated at over US $14 billion in the USA alone and over US $220 billion worldwide [50]. Yet ongoing colony losses in the USA and Europe defy causal explanation despite intensive research effort [51], [52] and identification of emergent and exotic pathogens [20]. Our discovery of a diverse and novel honey crop LAB microbiota common to all recognized honeybee species plus 3 stingless bee species may be the missing link in this worldwide problem. Since related microbiotas are found across bee species, it strongly suggests a close evolutionary relationship between bacteria and hosts, as well as underscoring the importance of LAB symbionts for bees. Not only are LAB symbionts involved in honeybee food production and preservation, they are also of importance in host defence against pathogen and transient microbes intercepted during foraging. The importance of this crop microbiota for honeybees is additionally strengthened by the fact that it is immediately transferred to the sterile crop of newly emerged bees by trophallactic exchange with nestmates.

Any beneficial effect from this microbiota may be undermined where prophylactic use of antibiotics is practiced (e.g. USA) or where their natural foodstuffs, honey and pollen, are supplemented by the beekeeper with synthetic sugars and pollen substitutes lacking LAB or their beneficial substances. The absence of LAB is especially problematic when the bees attempt to produce and preserve food for themselves and their brood, when feeding their brood with pollen lacking LAB or LAB derived antimicrobial substances, when nestmates establish a LAB microbiota in callows by trophallactic exchange, or when pathogens invade their hive. Emphasis now needs to be given to discovering the mechanisms of action of LAB against pathogens and food spoiling microbes, and how they can be used to resolve ongoing honeybee colony losses, in which LAB may be the important missing link. Altered beekeeping husbandry practices that enhance LAB are needed, or direct manipulation by supplementary feeding of individual or composite LAB members and their products could help alleviate CCD. Further functional analysis of LAB in bees will certainly enrich our understanding of insect-microbe symbioses and their evolutionary dynamics within complex eusocial insect societies.

## Materials and Methods

### Ethics

No specific permits were required for the described field studies. Local colleagues (described in the acknowledgments) collected samples where permission was not required i.e. not from nature reserves or privately-owned locations. The field studies did not involve endangered or protected species.

### LAB diversity in bees

We sampled the honey crop LAB microbiota of all 9 well recognized honeybee (Apini) species plus 3 stingless bee species (Meliponini). *Apis andreniformis* (n = 3 colonies), *Apis cerana* (n = 2 colonies), *Apis koschevnikovi* (n = 3 colonies), *Apis nuluensis* (n = 1 colony) and *Apis dorsata* (n = 1 colony) were collected from Borneo (Malaysia), *Apis laboriosa* (n = 2 colonies) from Nepal, *Apis florea* (n = 1 colony) from Thailand, *Apis nigrocincta* (n = 1 colony) from Indonesia, and *A. mellifera* (n = 25 colonies) from Sweden and Kenya. Samples of the stingless bee genera *Trigona* were collected from Thailand (n = 1 colony) and Borneo (n = 1 colony) (Malaysia), *Meliponula bocandeei* (n = 2 colony) from Kenya, and *Melipona beecheii* (n = 2 colonies) from Mexico. For each bee species, the honey crop LAB content of 10–20 bees was analysed. For *A. nuluensis* and *A. dorsata* we only analysed the honey crop content and the corbicular pollen from field collected bees as we were unable to sample colonies of these free-living bees. Isolation of LAB from honey crops, fresh honey, corbicular bee pollen and bee bread was performed as previously described [16], [19]. PCR-amplification of isolates for 16S rRNA gene sequencing, identification and phylogenetic analysis were performed according to Olofsson and Vásquez [16]. Additionally, the 16S rRNA gene sequences were also checked against the software RDP (Ribosomal Database Project II), accessible from the homepage (http://rdp.cme.msu.edu/). A total of 750 lactic acid bacterial isolates were retrieved in this study.

### Ontogeny of LAB

To determine how the crop microbiota is acquired, we marked Western honeybees (*A. mellifera*) at eclosion from their wax brood cells (n = 30), returned them to the hive, collected them at different ages, cultivated the contents of their crops and identified the LAB (cultivation and identification as described previously [16]).

### Maintenance of the honey crop microbiota

We performed *in vitro* and *in vivo* investigations of the symbionts with SEM and fluorescence microscopy. The SEM samples were prepared by freeze drying [53] and pictures were taken by Photographer Lennart Nilsson (Sweden).

The preparation and confocal fluorescence microscopy of bacteria in the honey crop was achieved as follows. Honeybees were fed with a mixture of honey and water containing (Sytox®, Green dye and BacLight™, Red bacterial stain, Molecular Probes) to discriminate the cells of the bee from the living bacteria. Following an incubation of approx. 15 min, the honey crop was dissected at room temperature. The crop was opened with a single longitudinal cut. To prevent the contraction of the muscles of the crop wall, the crop was rinsed in phosphate buffered saline (PBS) supplemented with 1 mM EDTA and mounted on a glass slide. Slides were examined using a TCS SP5 laser confocal microscope equipped with continuous spectrum white laser (Leica, Mannheim Germany). The images were captured using a 63× oil immersion objective (NA 1.4 HCX PL APO CS) with filter setup adapted to FITC and Texas Red dual colour illumination. The raw images were processed in ImageJ (NIH, Bethesda, USA) using median filtering. The Z-stacks was visualized using the ImageJ plugin 3D-viewer. The 3D-Movies (Video S1 and Video S2) show a projection of 90 confocal z-sections through a z-depth of 45.3 µm, covering an area of 246×246 µm in the xy-direction.

### *Melissococcus plutonius* bioassay

We investigated possible inhibitory effects on European foulbrood (EFB), a major bacterial pathogen of larval honeybees, from the LAB microbiota using both *in vitro* and *in vivo* tests, as previously described [24]. As adult honeybee workers feed larvae with crop contents, this represents a typical means by which larval food acquires LAB. Bacterial suspensions of *Melissococcus plutonius* (provided by Dr. Jean-Daniel Charrière, Agaroscope, Switzerland, Accession nr: JN689233) were prepared fresh for each experiment and diluted in larval food for final concentrations of 10^7^,10^6^ and 10^5^ bacteria per ml. A mixture of the thirteen previously described honeybee LAB [16], [17], [18] in approximately equal proportions was diluted in larval food for a final, total concentration of 10^7^ LAB per ml. *A. mellifera* worker larvae were grafted and reared *in vitro* [24]. Briefly, first instar worker larvae were transferred to the surface of the larval diet of the different treatments. i) control group provided with uninfected diet, ii) control group initially fed uninfected diet but LAB supplemented food after 48 hours onwards and iii) experimental groups provided larval diet spiked with defined amounts of *M. plutonius* (10^7^,10^6^ and 10^5^ bacteria ml^−1^). Twenty-four hours post exposure; larvae were transferred to wells containing uninfected diet and LAB supplemented food 48 hours post-infection onwards. The experiment was finished 21 days post-infection and larval mortality was monitored daily. A total of 420 larvae were used in two replicate experiments. The PoloPlus Probit and Logit Analysis program (version 2.0, LeOra software) was used to compare mortality rates between the experimental groups in the exposure bioassay.

### Flowers, nectar, pollen and microorganisms

We analysed the microbial composition of 15 flowers frequently visited by *A. mellifera* in Sweden (Table S1). Flowers were collected aseptically in Kullaberg, Sweden. The flowers were then shaken in sterile buffer (PBS) and immediately transported to the Laboratory at Lund University. Dilutions were made with sterile peptone water (0.2% w/v), spread on MRS (Oxoid), APT (Oxoid) and TSB (Oxoid) agar plates incubated anaerobically at 35°C (MRS and APT agar plates) and aerobically at 22°C (TSB agar plates) during 5 days. Identification of the microbial isolates was achieved by sequencing the 16S rRNA genes (for bacteria) [16] and the D1-D2 regions of the LSU 26S rRNA genes (for yeasts) [54].

### Dual-culture overlay assay

We analysed the inhibition properties of all honey crop LAB grown individually and together against the pathogen *M. plutonius* and also against the 55 bacterial strains and 5 yeast strains isolated from flowers. The assays were performed as earlier described [55] with the following modifications. LAB were inoculated on MRS agar plates (Oxoid, supplemented with 0.1% L-cysteine and 2.0% fructose) during 12 hours. We used the medium previously described for cultivation of *M. plutonius* [56], [57] and the same media as for the isolation of microbes from flowers for the over layer of soft agar. After an incubation of 2–4 days depending on growth rates the zone of inhibition was measured.

### LAB and antibiotics

Honey crop LAB were tested for susceptibility to oxytetracycline and tylosin by disc diffusion on MRS agar (Oxoid, supplemented with 0.1% L-Cysteine and 2.0% fructose).

## Supporting Information

Table S1 **The microbial composition of the flowers of 15 Angiosperma (left column) frequently visited by** ***Apis mellifera*** **bees in Sweden.** Fifty five different species of bacteria and 5 yeast species from the flowers were analysed *in vitro* against all 13 honey crop LAB from *A. mellifera* grown individually and together (right column). Inhibition zones are displayed as diameter in millimetres. Zero indicates no inhibition and (-) indicates no result.
(DOC)

Video S1 **A biofilm formed by the LAB microbiota is seen as a red carpet and the confocal z-stack reveals that the bacteria are located in crypts all through the z-depth of the crop wall.**
(AVI)

Video S2 **A 3D-view of a typical biofilm is shown in a 360 degree rotation of a projection of 90 confocal z-sections through a z-depth of 45.3 µm.**
(AVI)
