# Correction: Symbionts as Major Modulators of Insect Health: Lactic Acid Bacteria and Honeybees

**DOI:** 10.1371/annotation/3ac2b867-c013-4504-9e06-bebf3fa039d1

**Published:** 2012-07-12

**Authors:** Alejandra Vásquez, Eva Forsgren, Ingemar Fries, Robert J. Paxton, Emilie Flaberg, Laszlo Szekely, Tobias C. Olofsson

Information is missing from the Competing Interests statement. The full statement should read: "Alejandra Vasquez and Tobias Olofsson are the founders of and hold stocks in ConCellae AB, a biotech company that develops and markets functional food products, including honey-based products. Alejandra Vasquez and Tobias Olofsson are inventors and owners of a patent application related to the applications of *Lactobacillus* and *Bifidobacterium* strains isolated from honey. The study was not funded by ConCellae AB." 

