# Supplementary material for: Symbionts as Major Modulators of Insect Health: Lactic Acid Bacteria and Honeybees
Source: PLoS One. 2012 Mar 12;7(3):e33188. doi: 10.1371/journal.pone.0033188 (PMC3299755; doi:10.1371/journal.pone.0033188)
Supplement: Table S1 — The microbial composition of the flowers of 15 Angiosperma (left column) frequently visited by Apis mellifera bees in Sweden. Fifty five different species of bacteria and 5 yeast species from the flowers were analysed in vitro against all 13 honey crop LAB from A. mellifera grown individually and together (right column). Inhibition zones are displayed as diameter in millimetres. Zero indicates no inhibition and (-) indicates no result. (DOC) [file pone.0033188.s001.doc]

**Table S1. Inhibition tests of honey crop LAB against microbes from flowers.**

| **Isolates of bacteria and yeast^a^** | **Sampled flower^b^** | **Most closely related type strain^c^** | **Compared sequence length and % similarity^d^** | **Bin7** | **Bin2** | **Hma3** | **Bma6** | **Hon2** | **Hma11** | **Bin4** | **Fhon13** | **Fhon2** | **Hma8** | **Bma5** | **Biut2** | **Hma2** | **All 13** |
| --- | --- | --- | --- | --- | --- | --- | --- | --- | --- | --- | --- | --- | --- | --- | --- | --- | --- |
| ljubL1r (JN167926) | *Calluna vulgaris* (Heather) | *Lactobacillus kunkeei* YH-15^T^ (Y11374) | 832 (93.4) | 0 | 0 | 0 | 0 | 0 | 0 | 11 | 9 | 12 | 0 | 0 | 0 | 0 | 13 |
| ljubL4to (JN167928) | *Calluna vulgaris* (Heather) | *Lactobacillus concavus* AS 1.5017^T^ (AY683322) | 768 (88.3) | 0 | 0 | 0 | 0 | 0 | 8 | 0 | 7 | 8 | 0 | 0 | 0 | 0 | 12 |
| ljubL4ap (JN167933) | *Calluna vulgaris* (Heather) | *Leuconostoc pseudomesenteroides* NRIC 1777^T^ (AB023237) | 827 (99.2) | 0 | 0 | 0 | 0 | 10 | 0 | 7 | 0 | 8 | 0 | 0 | 0 | 0 | 12 |
| ljubL3ap (JN167935) | *Calluna vulgaris* (Heather) | *Leuconostoc holzapfelii* LMG 23990^T^ (AM600682) | 777 (99.6) | 0 | 0 | 0 | 0 | 6 | 8 | 6 | 10 | 11 | 0 | 0 | 0 | 0 | 15 |
| ljubL8ts (JN167963) | *Calluna vulgaris* (Heather) | *Asaia siamensis* S60-1^T^ (AB035416) | 860 (99.3) | 0 | 0 | 0 | 0 | 21 | 0 | 15 | 0 | 16 | 0 | 0 | 0 | 0 | - |
| smltbL2ap (JN167929) | *Fragaria vesca* (Wild strawberry) | *Carnobacterium gallinarum* DSM 4847^T^ (AJ387905) | 875 (98.7) | 6 | 7 | 8 | 8 | 0 | 13 | 0 | 19 | 9 | 0 | 7 | 8 | 8 | 26 |
| smltbL5ts (JN167961) | *Fragaria vesca* (Wild strawberry) | *Variovorax boronicumulans* BAM-48^T^ (AB300597) | 794 (98.7) | 20 | 20 | 22 | 18 | 20 | 30 | 30 | 38 | 36 | 12 | 12 | 14 | 18 | - |
| smltbL10ts (JN167962) | *Fragaria vesca* (Wild strawberry) | *Sphingomonas aurantiaca* MA101b^T^ (AJ429236) | 829 (99.9) | - | - | - | - | 17 | 10 | 10 | 0 | 12 | - | - | - | - | 17 |
| smltbL9ts (JN167957) | *Fragaria vesca*  (Wild strawberry) | *Pseudomonas agarici* LMG 2112^T^ (Z76652) | 844 (98.9) | 6 | 7 | 6 | 6 | 0 | 20 | 0 | 11 | 16 | 0 | 0 | 0 | 0 | 19 |
| murgbL4ap (JN167931) | *Hedera helix* (Ivy) | *Leuconostoc fallax* DSM 20189^T^ (AF360738) | 559 (96.6) | 0 | 0 | 0 | 0 | 0 | 12 | 0 | 11 | 11 | 0 | 0 | 0 | 0 | 10 |
| murgBL2ap (JN167932) | *Hedera helix* (Ivy) | *Fructobacillus fructosus* DSM 20349^T^ (AF360737) | 842 (99.5) | 0 | 0 | 0 | 0 | 0 | 13 | 7 | 14 | 14 | 0 | 0 | 0 | 0 | 11 |
| murgBL2to (JN167936) | *Hedera helix* (Ivy) | *Fructobacillus pseudoficulneus* LC51^T^ (AY169967) | 633 (92.3) | 6 | 0 | 6 | 5 | 0 | 10 | 0 | 10 | 12 | 0 | 0 | 0 | 0 | 12 |
| murgBL2ts (JN167940) | *Hedera helix* (Ivy) | *Erwinia tasmaniensis* Et1/99T (AM055716) | 766 (97.8) | 17 | 13 | 12 | 12 | 14 | 24 | 8 | 25 | 25 | 7 | 8 | 15 | 10 | 25 |
| murgBL3ts (JN167968) | *Hedera helix* (Ivy) | *Curtobacterium flaccumfaciens* LMG 3645^T^ (AJ312209) | 884 (99.7) | 9 | 9 | 9 | 12 | 8 | 16 | 0 | 28 | 30 | 0 | 12 | 13 | 7 | 28 |
| vpabL2to (JN167927) | *Pyrus communis* (Pear) | *Lactobacillus lindneri* DSM 20690^T^ (X95421) | 785 (99.7) | 0 | 0 | 0 | 0 | 0 | 20 | 0 | 8 | 10 | 7 | 11 | 9 | 9 | 13 |
| vpabLja1a (JN167985) | *Pyrus communis* (Pear) | *Metschnikowia pulcherrima* NRRL Y-7111^T^ (U45736) | 446 (97.3) | 0 | 0 | 0 | 0 | 0 | 7 | 0 | 0 | 8 | 0 | 0 | 0 | 0 | 8 |
| blhbL1r (JN167934) | *Rubus caesius* (Dewberry) | *Leuconostoc mesenteroides* ATCC 8293^T^ (CP000414) | 856 (99.6) | 0 | 0 | 0 | 0 | 9 | 0 | 8 | 0 | 10 | 0 | 0 | 0 | 0 | 10 |
| blhbL8ap (JN167939) | *Rubus caesius* (Dewberry) | *Erwinia persicina* ATCC 35998^T^ (U80205) | 802 (99.0) | 11 | 13 | 11 | 0 | 9 | 20 | 8 | 22 | 22 | 8 | 16 | 7 | 13 | 16 |
| blhbL2ts (JN167945) | *Rubus caesius* (Dewberry) | *Cedecea davisae* DSM 4568^T^ (AF493976) | 845 (99.3) | 6 | 0 | 0 | 0 | 6 | 17 | 0 | 18 | 23 | 5 | 7 | 8 | 7 | 28 |
| blhbL4ap (JN167945) | *Rubus caesius* (Dewberry) | *Yersinia kristensenii* ATCC 33638^T^ (AF366381) | 838 (99.3) | 0 | 0 | 0 | 0 | 11 | 29 | 0 | 20 | 24 | 7 | 11 | 0 | 9 | 25 |
| blhabL5ts (JN167959) | *Rubus caesius* (Dewberry) | *Acinetobacter johnsonii* ATCC 17909^T^ (Z93440) | 836 (95.3) | 0 | 0 | 0 | 0 | 0 | 11 | 0 | 12 | 14 | 10 | 14 | 10 | 13 | 17 |
| bl_hBL9ts (JN167973) | *Rubus caesius* (Dewberry) | *Micrococcus luteus* DSM 20030^T^ (AJ536198) | 802 (100) | 11 | 14 | 18 | 12 | 10 | 18 | 7 | 20 | 29 | 8 | 9 | 10 | 9 | 40 |
| hallbL2ap (JN167938) | *Rubus idaeus* (Raspberry) | *Erwinia rhapontici* DSM 4484^T^ (AJ233417) | 846 (99.5) | 10 | 8 | 9 | 9 | 8 | 15 | 0 | 22 | 32 | 0 | 0 | 0 | 0 | 22 |
| hallbL4ap (JN167949) | *Rubus idaeus* (Raspberry) | *Pseudomonas flectens* ATCC 12775^T^ (AB021400) | 890 (96.1) | 0 | 0 | 0 | 0 | 0 | 22 | 0 | 18 | 22 | 9 | 10 | 9 | 9 | 14 |
| hallbL9ts (JN167960) | *Rubus idaeus* (Raspberry) | *Xanthomonas arboricola* LMG 747^T^ (Y10757) | 918 (99.2) | 7 | 5 | 5 | 5 | 0 | 0 | 0 | 0 | 16 | 0 | 0 | 7 | 7 | 18 |
| hallbL5ts (JN167974) | *Rubus idaeus* (Raspberry) | *Kocuria marina* KMM 3905^T^ (AY2113859) | 737 (99.7) | 10 | 0 | 5 | 7 | 0 | 13 | 0 | 16 | 23 | 0 | 0 | 0 | 0 | 42 |
| rspbjbbLj5 (JN167982) | *Rubus radula* (Raspberry) | *Aureobasidium pullulans* CBS 100524^T^ (FJ150952) | 519 (100) | 0 | 0 | 0 | 0 | 0 | 0 | 14 | 12 | 11 | 0 | 0 | 0 | 0 | 10 |
| rspbjbbLj3 (JN167981) | *Rubus radula* (Raspberry) | *Rhodotorula fujisanensis* CBS4551^T^ (AF189928) | 536 (100) | 0 | 0 | 0 | 0 | 0 | 14 | 0 | 15 | 20 | 0 | 0 | 0 | 0 | 9 |
| vitkbL6ap (JN167941) | *Trifolium repens* (White clover) | *Pantoea agglomerans* DSM 3493^T^ (AJ233423) | 914 (98.2) | 13 | 11 | 11 | 9 | 11 | 20 | 8 | 24 | 42 | 10 | 8 | 10 | 6 | 30 |
| vitkbL1ap (JN167947) | *Trifolium repens* (White clover) | *Serratia grimesii* DSM 30063^T^ (AJ233430) | 861 (98.7) | 8 | 0 | 5 | 10 | 6 | 12 | 0 | 12 | - | 0 | 0 | 0 | 6 | 22 |
| vitkbL3tsb (JN167954) | *Trifolium repens* (White clover) | *Pseudomonas graminis* DSM 11363^T^ (Y11150) | 849 (100) | 8 | 10 | 10 | 10 | - | - | - | - | 22 | 0 | 0 | 0 | 0 | - |
| vitklbL9ts (JN167979) | *Trifolium repens* (White clover) | *Pseudomonas trivialis* DSM 14937^T^ (AJ492831) | 731 (100) | 12 | 13 | 11 | 11 | 11 | 23 | 30 | 30 | 32 | 30 | 30 | 30 | 30 | - |
| vitkbL11ts (JN167965) | *Trifolium repens* (White clover) | *Agreia pratensis* P 229/10^T^ (AJ310412) | 745 (99.7) | 20 | 14 | 21 | 20 | 30 | 30 | 30 | 30 | 40 | 16 | 14 | 16 | 15 | 30 |
| maskros1ap (JN167937) | *Taraxacum* sect*. Hamata* (Dandelion) | *Erwinia persicina* ATCC 35998^T^ (U80205) | 865 (98.7) | - | - | - | - | - | - | - | - | - | 0 | 6 | 10 | 10 | - |
| mskroBL2a (JN167951) | *Taraxacum* sect*.Hamata* (Dandelion) | *Pseudomonas veronii* CIP 104663^T^ (AF064460) | 844 (99.9) | - | - | - | - | - | - | - | - | 32 | - | - | - | - | 38 |
| mskrsBL7ts (JN167952) | *Taraxacum* sect. *Hamata* (Dandelion) | *Pseudomonas cichorii* LMG 2162^T^ (Z76658) | 509 (98.6) | 15 | 8 | 7 | 7 | 8 | 8 | 0 | 9 | - | - | - | - | - | - |
| maskbL1to (JN167944) | *Taraxacum* sect. *Hamata* (Dandelion) | *Raoultella planticola* ATCC 33531^T^ (AF129443) | 570 (97.0) | 12 | 8 | 8 | 11 | 11 | 22 | 0 | 26 | 16 | 0 | 7 | 8 | 9 | - |
| maskbL4tsb (JN167956) | *Taraxacum* sect. *Hamata* (Dandelion) | *Pseudomonas abietaniphila* ATCC 700689^T^ (AJ011504) | 817 (97.7) | 18 | 17 | 11 | 12 | 0 | 32 | 12 | 43 | - | 10 | 10 | 11 | 6 | 39 |
| maskbL8ts (JN167971) | *Taraxacum* sect. *Hamata* (Dandelion) | *Microbacterium hominis* DSM 12509^T^ (AM181504) | 597 (97.2) | 12 | 8 | 9 | 11 | 19 | 32 | 32 | 22 | 24 | 5 | 8 | 8 | 7 | 11 |
| mskroBL6ap (JN167975) | *Taraxacum* sect. *Hamata* (Dandelion) | *Sanguibacter inulinus* ST50^T^ (X79451) | 879 (99.5) | 18 | 18 | 22 | 16 | 14 | 32 | 12 | 32 | 36 | 12 | 13 | 10 | 8 | 26 |
| mskroBL5ap (JN167958) | *Taraxacum* sect. *Hamata* (Dandelion) | *Pseudomonas umsongensis* Ps 3-10^T^ (AF468450) | 879 (98.5) | 8 | 12 | 8 | 5 | 12 | 26 | 10 | 23 | 28 | 18 | - | 18 | - | - |
| slg7bF (JN167950) | *Salix caprea*  (Goat Willow) | *Pseudomonas cannabina* CFBP 2341^T^ (AJ492827) | 780 (99.6) | 6 | 6 | 7 | 12 | 32 | 26 | 7 | 24 | 31 | 24 | 16 | 18 | 22 | - |
| slg2bF (JN167955) | *Salix caprea*  (Goat Willow) | *Pseudomonas koreensis* Ps 9-14^T^ (AF468452) | 802 (97.8) | - | 14 | - | 14 | - | - | - | 8 | 48 | - | - | - | - | - |
| slg1bF (JN167964) | *Salix caprea*  (Goat Willow) | *Frigoribacterium faeni* 801^T^ (Y18807) | 882 (99.5) | 28 | 18 | 18 | 22 | 24 | 40 | 30 | 48 | 30 | - | - | - | - | 39 |
| slg5bF (JN167967) | *Salix caprea*  (Goat Willow) | *Frondihabitans australicus* E1HC-02^T^ (DQ525859) | 876 (97.0) | 12 | 10 | 7 | 12 | 10 | 16 | 7 | 16 | 23 | 9 | 13 | 12 | 13 | - |
| ralrobL8ts (JN167942) | *Epilobium angustifolium* (Rosebay Willowherb) | *Pantoea ananatis* ATCC 33244^T^ (U80196) | 734 (99.9) | 13 | 10 | 11 | 13 | 15 | 25 | 10 | 34 | 34 | 0 | 0 | 0 | 0 | 30 |
| ralrobL1ts (JN167966) | *Epilobium angustifolium* (Rosebay Willowherb) | *Lactococcus lactis* NCDO 607^T^ (AB100802) | 912 (91.8) | 5 | 6 | 5 | 5 | 7 | 17 | 0 | 14 | 18 | 0 | 5 | 5 | 6 | 14 |
| ralroja3 (JN167980) | *Epilobium angustifolium* (Rosebay Willowherb) | *Metschnikowia reukaufii* NRRL Y-7112^T^ (U44825) | 454 (99.6) | 0 | 0 | 0 | 0 | 0 | 0 | 0 | 0 | 7 | - | - | - | - | 7 |
| blklbL1to (JN167948) | *Centaurea cyanus* (Cornflower) | *Brenneria quercina* DSM 4561^T^ (AJ233416) | 722 (97.1) | 9 | 7 | 7 | 9 | 10 | 26 | 12 | 32 | 32 | 0 | 0 | 7 | 0 | 30 |
| sktrBL10ts (JN167969) | *Lonicera xylosteum* (Fly Honey-suckle) | *Plantibacter flavus* P297/02^T^ (AJ310417) | 820 (99.6) | 12 | 8 | 8 | 8 | 7 | 9 | 6 | 14 | 21 | 0 | 8 | 8 | 8 | - |
| sktrBL11ts (JN167970) | *Lonicera xylosteum* (Fly Honey-suckle) | *Microbacterium hydrocarbonoxydans* DSM 16089^T^ (AJ698726) | 690 (96.8) | 20 | 16 | 15 | 20 | 16 | 33 | 9 | 25 | 33 | 7 | 9 | 11 | 5 | 38 |
| sktrbL8ts (JN167972) | *Lonicera xylosteum* (Fly Honey-suckle) | *Microbacterium profundi* Shh49^T^ (EF623999) | 511 (98.0) | 13 | 13 | 15 | 13 | 13 | 23 | 12 | 23 | 36 | 0 | 8 | 8 | 14 | 26 |
| sktrbl7ts (JN167976) | *Lonicera xylosteum* (Fly Honey-suckle) | *Rhodococcus erythropolis* DSM 43066^T^ (X79289) | 585 (99.9) | 10 | 0 | 0 | 0 | 6 | 16 | 9 | 14 | 22 | 0 | 0 | 0 | 0 | 19 |
| sktbL13ts (JN167977) | *Lonicera xylosteum* (Fly Honey-suckle) | *Rhodococcus globerulus* DSM 4954^T^ (X80619) | 698 (99.4) | 20 | 9 | 7 | 9 | 0 | 20 | 0 | 26 | 40 | 16 | 12 | 20 | 12 | 21 |
| sktrBL12ts (JN167978) | *Lonicera xylosteum* (Fly Honey-suckle) | *Pedobacter heparinus* DSM 2366^T^ (AJ438172) | 777 (97.8) | 20 | 15 | 14 | 14 | 16 | 24 | 0 | 26 | 37 | 8 | 8 | 9 | 0 | 32 |
| grvapbL4ap (JN167943) | *Malus domestica* (Apple) | *Pantoea agglomerans* DSM 3493^T^ (AJ233423) | 820 (96.8) | 14 | 14 | 13 | 9 | 42 | 18 | 28 | 20 | 33 | 0 | 0 | 7 | 7 | 47 |
| grvapbL4ts (JN167953) | *Malus domestica* (Apple) | *Pseudomonas rhizosphaerae* IH5^T^ (AY152673) | 794 (97.7) | - | - | - | - | 0 | 0 | 10 | 18 | 18 | - | - | - | - | 16 |
| rvabL2bts (JN167930) | *Malus domestica* (Apple) | *Bacillus simplex* DSM 1321^T^ (AJ439078) | 814 (99.6) | 0 | 0 | 0 | 0 | 0 | 15 | 0 | 11 | 15 | 12 | 13 | 14 | 16 | 14 |
| grvapbLja7 (JN167984) | *Malus domestica* (Apple) | *Cryptococcus victoria* CBS 8685^T^ (AF363647) | 544 (98.7) | 10 | 9 | 9 | 14 | 9 | 20 | 0 | 18 | 18 | 15 | 6 | 8 | 13 | - |
| skogslind 3 (JN167983) | *Tilia cordata*  (Small-Leaved Lime) | *Candida rancensis* CBS 8174^T^ (AJ508580) | 455 (100) | 0 | 0 | 0 | 0 | 0 | 8 | 0 | 10 | 11 | 0 | 0 | 0 | 0 | 9 |

^a^GenBank accession numbers are shown in parentheses (JN167926-JN167985).

^b^The flower names in Latin following their English names displayed in parentheses.

^c^GenBank accession numbers of type strains are shown in parentheses.

^d^The similarity to closest sequence match of bacterial and yeast type strains is shown within parentheses as a percentage of base pair matches in the respectively partial 16S rRNA and 26S rRNA genes, established by comparing the bacterial sequences in the database of Ribosomal Database project II (http://rdp.cme.msu.edu/) and the yeast sequences with BLAST (http://blast.ncbi.nlm.nih.gov/Blast.cgi) in GenBank.
